# Evaluation of two point-of-care molecular diagnostic platforms for rapid detection of equine Hendra virus

**DOI:** 10.1016/j.vas.2026.100713

**Published:** 2026-05-30

**Authors:** Lyndal Hulse, Leonard Izzard, Singanallur Balasubramanian Nagendrakumar, Axel Colling, Darren Underwood, Luke Driver, David T. Williams, Benjamin Ahern

**Affiliations:** a School of Veterinary Science, The University of Queensland, Gatton QLD 4343, Australia; b CSIRO, Australian Centre for Disease Preparedness, Geelong VIC 3220, Australia; c Biosecurity Sciences Laboratory, Department of Primary Industries, Coopers Plains, QLD 4108, Australia; d World Organization for Animal Health Collaborating Centre for Diagnostic Test Validation Science in the Asia-Pacific Region, CSIRO, Australian Centre for Disease Preparedness, Geelong VIC 3220, Australia

**Keywords:** Validation, Point-of-care-test, Hendra virus, Horses, Henipavirus

## Abstract

Hendra virus (HeV) is a lethal zoonotic pathogen endemic to eastern Australia, posing significant risks to equine and human health. Rapid field detection of HeV enables timely intervention and outbreak management. This study evaluated candidate point-of-care (POC) molecular diagnostic platforms for HeV detection in equine samples: including a loop-mediated isothermal amplification (DARQ RT-LAMP) assay and real-time reverse transcription quantitative polymerase chain reaction (RT-qPCR). Comparative analytical evaluation demonstrated that RT-qPCR exhibited superior analytical sensitivity relative to DARQ RT-LAMP, with a limit of detection of 1 copy/µL compared with 1,000 copies/µL, respectively. On this basis, DARQ RT-LAMP was not progressed beyond initial analytical evaluation due to insufficient sensitivity for the intended application. Bayesian Latent Class Model (BLCM) analysis estimated diagnostic sensitivity of 63.1% (95%PI 48.8–76.1%) and 80.4% (95%PI 67.6–90.2%) for RT-qPCR with HUDSON-prepared samples and extracted RNA, respectively, with identical specificity of 96.5% (95%PI 85.9–99.9%) for both sample types in virus transport medium. In 10% EDTA blood, diagnostic sensitivity was 71.3% (95%PI 54.2–85.1%) and 88.3% (95%PI 74.2–97.3%), respectively, with comparable specificity. Preliminary assessment of repeatability and reproducibility was promising (CVs <10%), although further field studies are required. These findings demonstrate that RT-qPCR, combined with rapid HUDSON sample preparation, provides a feasible molecular POC approach for preliminary rule-in or exclusion of HeV infection in horses while confirmatory laboratory testing is pending, supporting early risk management and reduced occupational exposure.

## 1 Introduction

Hendra virus (HeV) is a large, pleomorphic enveloped RNA virus belonging to the genus *Henipavirus*, and family *Paramyxoviridae*. HeV is a highly lethal zoonotic pathogen that poses a serious public and veterinary health concern in eastern Australia. High mortality has been observed in a number of spillover events from its natural reservoir *Pteropus* fruit bat species (flying foxes; family *Pteropodidae*) to horses (Halpin et al., 2011). HeV is also transmissible to humans from horses with very high fatality rates in confirmed equine cases across Queensland and New South Wales since its emergence in 1994 (Young, Selvey, & Symons, 2011). The most recent spillover event occurred with the infection of an unvaccinated horse occurring in July 2025 on a property in Southeast Queensland, Australia. Periodic virus transmission from flying foxes to horses potentially exposes veterinarians, veterinary clinic staff and horse handlers to this Risk Group 4 pathogen, the highest classification issued by the World Health Organisation, and reflects the importance of HeV as a seriously dangerous pathogen requiring the highest level of human protection.

Naturally occurring HeV infection can cause a wide range of clinical signs in horses due to the virus targeting endothelial cells. Early signs can include fever, increased heart rate and restlessness. Other common features may include difficulty breathing, weakness and neurological signs such as uncoordinated gait and muscle twitching, quickly leading to death in most cases (Middleton, 2014). Because the clinical signs associated with HeV infection are not pathognomonic, laboratory confirmation of HeV infection is made by detection of HeV nucleic acid primarily in blood, nasal, oral and rectal swabs using molecular diagnostic techniques such as real-time reverse transcription quantitative polymerase chain reaction (RT-qPCR).

In addition to molecular diagnostics, serological assays such as enzyme-linked immunosorbent assays (ELISA) have been used in HeV investigations to assess prior exposure (McNabb et al., 2025). However, these approaches do not detect active infection during the acute clinical phase and are therefore not suitable for diagnostic decision making in suspected HeV cases. As a result, RT-qPCR remains the primary method for confirmation of acute HeV infection in horses.

Current HeV RT-qPCR methods (Annand etal., 2021; Wang et al., 2021; WOAH, 2023a) are required to be performed in laboratory settings by highly trained molecular laboratory professionals and samples must be transported under the appropriate conditions from the point of collection to the laboratory. Due to the cost and, at times, logistically difficult sample transport options, laboratory diagnosis or confirmation of HeV infection may be delayed, consequently delaying veterinary treatment, which becomes a major welfare issue for the horse.

Over the past decade, loop-mediated isothermal amplification (LAMP) technology has become an established tool in molecular diagnostics, reflecting its rapid turnaround time, relatively high sensitivity and specificity, cost-effectiveness, and field applicability (Zhao, Chen, Li, Wang, & Fan, 2015). The detection amplification by release of quenching (DARQ) technique, which uses a quencher-modified primer and a fluorophore-labelled strand, facilitates real-time colour multiplexing in LAMP assays (Tanner, Zhang, & Evans, 2012). When comparing LAMP to a reference standard molecular assay such as RT-qPCR, is essential to assess specificity and sensitivity, particularly for samples with higher cycle threshold values (Moehling, Choi, Dugan, Salit, & Meagher, 2021). Furthermore, the availability of portable real-time PCR thermocyclers now provides a viable alternative platform to LAMP for point-of-care molecular diagnostics.

Although portable PCR platforms are increasingly used for field diagnostics, isothermal methods such as LAMP remain attractive due to their simplicity and minimal equipment requirements. Because no DARQ-based multiplex LAMP assay for HeV has previously been evaluated, we included a preliminary feasibility assessment of this method to determine whether its analytical performance met the minimum threshold required for further validation. This screening step was performed to assess whether a lower-complexity amplification system could realistically serve as a point-of-care alternative before progressing to full validation of the more analytically sensitive RT-qPCR platform.

For the development and validation of POC assays for Risk Group 4 pathogens, such as HeV, there are extra considerations regarding biosafety and biosecurity required particularly for assessment of robustness and ruggedness (Department of Agriculture, 2020; WOAH, 2023b). For instance, the use of inactivated virus or synthetic HeV nucleic acid for positive control material will avoid the limitations of working under BSL-4/PC4 conditions and facilitate inter-laboratory comparison testing. Moreover, for application in the field or veterinary clinic, it is optimal to incorporate a specimen inactivation method that significantly decreases the risk of pathogen exposure to the operator. In addition, the recent discovery of a novel HeV variant, designated genotype 2 (HeV g2) in flying foxes (Peel et al., 2022; Wang et al., 2021), and a fatal horse case (Annand et al., 2021; Taylor et al., 2022), highlights the need for diagnostic assays that can reliably detect both HeV genotypes. Therefore, a HeV POC test must allow simultaneous detection of both HeV g1 and g2, in addition to an internal PCR inhibition control within an individual reaction tube.

In the absence of specific validation standards for POC tests, information from science-based validation chapters such as the World Organisation for Animal Health (WOAH) Manual of Diagnostic Tests and Vaccines for Terrestrial Animals (WOAH, 2023b) and Subcommittee on Animal Health Laboratory Standards (SCAHLS) (Department of Agriculture, 2020) are suitable references to be adapted to specific needs of POC tests. Additionally, the test must be robust and rugged enough to perform consistently across various settings, from field conditions to veterinary clinics, while also being user-friendly for non-specialist operators with limited training and variation in proficiency. By meeting these external validation criteria, the assay addresses point-of-care specific parameters such as robustness and ruggedness and significantly contributes to the preliminary detection and management of HeV infection for equines.

For the purposes of this study, the intended clinical role of POC molecular testing for HeV was defined as a preliminary decision support tool to assist veterinarians with early case management, infection control and risk mitigation while confirmatory laboratory testing is pending. The assays evaluated here are not intended to replace reference laboratory diagnostics, but rather to provide rapid, on-site molecular evidence to support rule-in or exclusion decisions during the initial assessment of horses with clinical signs compatible with HeV infection.

Here, we report the laboratory-based evaluation of two molecular POC tests for the detection of HeV detection in equine samples: a novel DARQ RT-LAMP assay and a real-time RT-qPCR assay (Wang et al., 2021). For the validation of these tests, we defined their intended purpose as tests for the preliminary rule-in or exclusion of HeV infection in horses in the field or veterinary clinic. To further enhance the feasibility of POC HeV diagnostics, the HUDSON (heating unextracted diagnostic samples to obliterate nucleases) method (Myhrvold et al., 2018) for sample inactivation was adopted and incorporated into the workflow of these tests to ensure viral inactivation while preserving RNA integrity. Although the HUDSON workflow has been applied to several emerging human viral pathogens (Barnes et al., 2020; Myhrvold et al., 2018), its adaptation for HeV represents a distinct translational challenge because HeV is managed exclusively under PC4 conditions and is primarily encountered in veterinary rather than human diagnostic settings. Factors such as equine sample matrices, biosafety requirements for safe field-based inactivation, and the need for compatibility with portable, battery-operated platforms have not previously been evaluated. Accordingly, the present study provides the first assessment of HUDSON sample preparation for equine HeV diagnostics and its integration into a point-of-care workflow designed specifically to support rapid, preliminary on-site veterinary decision-making.

## 2 Materials and methods

### 2.1 HeV viral isolates, plasmids, virus culture, and RNA extraction

Genotype 1 (HeV-g1) Hendra virus/Australia/Horse/2008/Redlands, and Genotype 2 (HeV-g2) HeV-g2/Australia/Horse/2015/Gympie viral isolates used in this study were sourced from the Australian Centre for Disease Preparedness (ACDP) BSL-4 facility (CSIRO, Geelong Australia) and were treated with 50kGy gamma-irradiation for viral inactivation at Steritech (Dandenong, Australia). Plasmids containing fragments of the HeV P/V/C and M genes were constructed. Briefly, the HeV P/V/C clone was constructed as described in (Marsh et al., 2013), and the HeV M clone was constructed by amplifying the full open reading frame (ORF) of the HeV M gene via RT-PCR from RNA extracted from the supernatant of cells infected with the 1994 HeV isolate. The amplified M gene was subsequently cloned into the pCAGGS expression vector using SacI/XhoI restriction sites. Resulting plasmids containing P/V/C and M genes were purified using Maxiprep (Qiagen) according to the manufacturer's instructions., and sequences confirmed by Sanger sequencing.

Reference HeV stocks were cultured and titrated as previously described (Annand et al., 2021). Efficacy testing of the HUDSON method for inactivation of HeV was undertaken using Vero cells (ATCC #C1008) incubated in maintenance media consisting of Minimal Essential Media containing Earle’s salts (EMEM) supplemented with 2% Foetal Bovine Serum (FBS), 10mM HEPES, 2 mmol/L L- glutamine, 100 U/mL penicillin, 100 µg/ml streptomycin and 250 ng/ml amphotericin B (all sourced from Thermo Fisher Scientific, Australia).

RNA extraction from virus stocks and clinical specimens was performed using the MagMAX™ Pathogen RNA/DNA Kit (Thermo Fisher Scientific, Australia) and purified on the KingFisher™ Flex instrument (Thermo Fisher Scientific, Australia) as per manufacturer’s instructions.

### 2.2 Virus inactivation proof of concept using the HUDSON method

A simple and rapid heat inactivation protocol based on the HUDSON method (Myhrvold et al., 2018) was optimised for inactivation of HeV in equine clinical samples during template preparation. The sample inactivation buffer comprised 0.01M NaOH and 0.5M disodium EDTA (pH 12.0) adjusted to pH 8.7 with 0.1M Tris-HCl, diluted in distilled water. Samples were heat inactivated by incubation at 95°C for 5 min.

Although the HUDSON workflow has been applied to several human viral pathogens, its use for HeV diagnostics required evaluation under PC4 biosafety containment and with relevant equine sample matrices. The optimisation performed here therefore represents the first application of HUDSON to equine HeV diagnostics and was essential to determine whether rapid field-safe inactivation could be achieved without compromising RNA integrity for downstream POC testing.

Proof of effectiveness of the inactivation method was performed via virus viability testing at the ACDP BSL-4 facility. Briefly, whole blood or nasal swab in virus transport medium from a healthy horse that tested negative for HeV by RT-qPCR (Feldman et al., 2009) was spiked with a HeV isolate (Hendra virus/Australia/Horse/2008/Redlands) to a final concentration of 1 × 10^6^ TCID/mL Next, 100 µL of spiked sample was added to 100 µL of either inactivation buffer or phosphate buffered saline (PBS) (200µl total volume) and were incubated at either 95°C or room temperature (positive controls) for 5 min as described in Table 1. Following this, confirmation of inactivation was determined by cell culture challenge. Briefly, samples were purified post-treatment using ZEBA spin desalting columns 40kDa MWCO (Thermo Fisher Scientific, Australia) as per manufacturer’s recommendations, with 100 µL of purified sample and 900 µL of maintenance media added to each well of a 6 well plate containing a confluent Vero monolayer. Plates were incubated for 7 days, and the presence or absence of cytopathic effect (CPE) was determined. Wells containing no CPE were passaged an additional 2 times for 7 days and tested by HeV RT-qPCR to confirm absence of virus replication.

**Table 1:** Sample inactivation treatment groups.

Sample	Sample Treatment
HeV spiked equine nasal swab in virus transport medium diluted 1:10 with inactivation buffer	1. Incubation at 95°C for 5 min
	2. Incubation at room temperature
HeV spiked equine whole blood diluted 1:10 with inactivation buffer	1. Incubation at 95°C for 5 min
	2. Incubation at room temperature
HeV spiked whole blood diluted 1:10 in PBS (positive control)	1. Incubation at room temperature
HeV spiked virus transport medium diluted in PBS (positive control)	1. Incubation at room temperature
Negative control	Untreated inactivation buffer

### 2.3 POC HeV DARQ RT-LAMP and RT-qPCR primers

DARQ RT-LAMP multiplex primer sets were designed for simultaneous detection of HeV g1 or g2 corresponding to the P/V/C gene (GenBank: AF017149), and an internal control corresponding to the mitochondrial mammalian 16S rRNA gene (Cawthraw et al., 2009). Six oligonucleotide primers, consisting of the outer forward primer (F3), outer backward primer (B3), forward inner primer (FIP), backward inner primer (BIP), loop forward (FL), and loop backward (BL), were selected using LAMP Designer 1.15 software (Premier Biosoft). A Basic Local Alignment Search Tool (BLAST) search (http://blast. ncbi.nlm.nih.gov/Blast.cgi) was performed to confirm primer specificity. From each set of primers, both FIP primers (comprising F1c + F2 sequences) were labelled at 5’ end by adding a dark quencher (Q-FIP): Iowa Black FQ (IAbFQ) for HeV-FIP primer and Iowa Black RQ (IAbRQ) for internal control-FIP primer. F1c sequence complementary probes were designed and labelled with a fluorophore at 3’ end: 6-Carboxyfluorescein (6-FAM) and (5-TEX615) for amplification of HeV and the internal control, respectively. Comparison of DARQ RT-LAMP to RT-qPCR was performed using modified primer and probe sequences previously described (Wang et al., 2021), combined with an internal control targeting the mammalian mitochondrial 16S rRNA gene as described by (Cawthraw et al., 2009). This internal control was included to assess potential amplification inhibition associated with complex sample matrices such as whole blood and nasal swabs, with consistent amplification indicating minimal inhibition effects. RT-LAMP primers, Q-FIP primers duplexed to their complementary dye labelled probes and RT-qPCR primers were synthesised by Integrated DNA Technologies (Coralville, IA, USA). Table 2 provides DARQ RT-LAMP and RT-qPCR primer and probe information.

**Table 2:** DARQ RT-LAMP and RT-qPCR primer and probe sequences.

Testing Platform	Target	Primers	Sequence (5 → 3)	Reference
LAMP	HeV P/V/C gene	F3	YTGGATYTAGTYAATGATGGC	This study
		B3	GTATRTCATCTGRRYGYTCTGC	
		FIP	ACYGGTGCTCTGCAAGAAGTCTCAAGCATCCAACAACCAA	
		BIP	GGAGAACATGAACAGGCTGAGGACTGGRTAKATCCTCCACAYY	
		LF	CCATGCTYTTGTCCTGTCTT	
		LB	TGYCTAAGAATGATGGARGTAC	
		Q-FIP	**IAbFQ**-ACYGGTGCTCTGCAAGAAGTCTCAAGCATCCAACAACCAA	
		Probe	GACTTCTTGCAGAGCACCRGT-**6-FAM**	
	Mammalian 16S rRNA	F3	ACTGTCTCTTACTTCCAATCAG	This study
		B3	TCTGGATTTAAATCACTCGGAG	
		FIP	AAGCTCCATAGGGTCTTCTCGTTGAAATTGACCTTCCCGTG	
		BIP	ACCTTAACCTTCAGGGACAACATTGTTCTCCGAGGTCACC	
		LF	ATTTAGTCATTCCCGCCTCTT	
		LB	TGAATCAGCAATTTCGGTTGG	
		Q-FIP	**IAbRQ**-AAGCTCCATAGGGTCTTCTCGTTGAAATTGACCTTCCCGTG	
		Probe	ACGAGAAGACCCTATGGAGCTT-**TEX615**	
RT-qPCR	HeV M gene	Forward	CTGATCYACRTTRCGGCAAACCTT	(Wang et al., 2021)
		Reverse	GGCCCRCTTCAYCATCTCTTAC	
		Probe	**FAM**- CAGCATTGA/**ZEN**/ATATTGAYCCRCCAGTCA-**IBFQ**	
	Mammalian16S rRNA	Forward	AGGGATAACAGCGCAATC	(Cawthraw et al., 2009)
		Reverse	ATCGTTGAACAAACGAACC	
		Probe	**Cy5**-TTTACGACCTCGATGTTGGATC	

Although DARQ RT-LAMP assays offer an attractive low-complexity platform for POC diagnostics, no DARQ RT-LAMP assay for HeV had been previously evaluated. Therefore, DARQ RT-LAMP was included in this study solely as a preliminary feasibility assessment to determine whether its analytical performance met the minimum sensitivity required for progression to full validation. DARQ RT-LAMP was evaluated as part of a staged validation framework, whereby assays were required to meet predefined analytical sensitivity thresholds prior to progression to diagnostic and field validation.

### 2.4 POC HeV DARQ RT-LAMP and RT-qPCR Reactions

Set up of DARQ RT-LAMP reactions were based on original protocols previously described by and (Nanayakkara and White, 2019). Each DARQ RT-LAMP assay target was optimised for each modified primer set (incorporating the additional Q-FIP primers and the labelled probes into the master mix) using different reaction conditions. Simplex DARQ RT-LAMP reactions (i.e., including template from only one gene target, HeV P/V/C gene or Eq 16S mtDNA) were carried out in the presence of *Bst* 2.0WarmStart DNA Polymerase (*Bst* 2.0 WS) (NEW ENGLAND BIOLABS Ltd., Ipswich, UK) in a volume of 25 µL containing 1.4 mM of each dNTP (BIORON GmBH, Römerberg, Germany), 1X Amplification Buffer (20 mM Tris-HCl (pH 8.8), 50 mM KCl, 10 mM (NH_4_)_2_SO_4_, 2 mM MgSO4, 0.1% Tween20), 0.2 µM outer F3/B3 primers, 0.4 µM LF and LB, and 1.6 µM inner primer BIP and 1.6 µM of a mix including nonlabelled inner primer FIP and QPD (duplexed Q-FIP and labelled probe) and were tested with real-time fluorescence reading on the Genie III portable isothermal amplification instrument (OPTIGENE Ltd, Horsham, UK) with a reaction time of 65°C for 30 min. Primer concentrations and reaction conditions were optimised in singleplex DARQ RT-LAMP reactions prior to duplex implementation. Once singleplex DARQ RT-LAMP reactions were standardised, duplex DARQ RT-LAMP reactions (i.e., for simultaneous amplification of both target templates) were performed using a 50:50 proportion of primer sets for each gene target, with a final combined primer concentration of 3.6 µM.

RT-qPCR was performed in a 15 µL reaction volume containing 5 µL template, 7.5 μL of AgPath One-step RT-PCR buffer (Ambion, Texas, USA), 0.6 μL of 25 × reverse transcriptase, 0.3 μL of 45 μM each HeV primer, 0.3 μL of 12.5 μM HeV TaqMan probe, 0.15 µL of 20 µM each 16S rRNA primers, 0.15 µL of 5 µM mtDNA TaqMan probe and 0.55 μL of nuclease free water. The RT-qPCR assays were performed under the following conditions: 10 min at 45°C for reverse transcription of RNA, 10 min at 95°C for inactivation of reverse transcriptase, followed by 40 cycles of 95°C for 15 s, 60°C for 45 s using a Mic PCR cycler (Bio Molecular Systems, Upper Coomera, Australia). An RT-qPCR cycle threshold (Ct) value of < 40 was used to define a positive result for all RT-qPCR analyses, consistent with established reference laboratory diagnostic criteria.

### 2.5 Validation of POC tests

Information from science-based validation chapters such as the WOAH Manual of Diagnostic Tests and Vaccines for Terrestrial Animals (WOAH, 2023b) and SCAHLS, (Department of Agriculture, 2020) were adapted for application to the validation of the HeV DARQ RT-LAMP and RT-qPCR assays. For this study, we defined the intended purpose as a point-of-care nucleic acid detection test for the preliminary rule-in or exclusion of HeV in horses in the field or veterinary clinic. Consistent with this intended purpose, validation focused on analytical and diagnostic performance parameters relevant to preliminary molecular screening, rather than replacement of laboratory based confirmatory testing.

Test validation is an incremental process and comprises five stages in the assay validation pathway; 1) assay analytical characteristics (sensitivity, [ASe] and specificity, [Asp] and repeatability); 2) assay diagnostic characteristics (cut-off thresholds, sensitivity [DSe], and specificity, [DSp]); 3) assay reproducibility; 4) assay implementation; and 5) monitoring assay performance following validation and implementation (WOAH, 2023b). In this study, we undertook stages 1 and 2, and preliminary stage 3 of the assay validation pathway.

*Stage 1: Assay Validation - Analytical Characteristics*

Analytical specificity (ASp) was assessed by testing a total of 23 purified viral and bacterial strains or isolates, representative of equine pathogens that may be expected to be included in a differential diagnosis of acute equine neurological disease, with HeV duplex DARQ RT-LAMP and RT-qPCR (Supp. Table 1).

Analytical sensitivity (ASe), synonymous with limit of detection (LOD), was evaluated with both tests using duplicate 10-fold serial dilutions of inactivated HeV g1 gene plasmids. Reactions contained the equivalent of 10^8^ to 1 gene copies per µL of the M gene (RT-qPCR), and 10^5^ to 1 gene copies per µL of the P/V/C gene (DARQ RT-LAMP).

Within-run repeatability (intra-assay) of the HeV duplex DARQ RT-LAMP and RT-qPCR assays was estimated by evaluating the percent Coefficient of Variation (CV%) in results of replicates. Serially diluted gamma-irradiated HeV g1 and g2 isolates were spiked into sample inactivation buffer and prepared via the HUDSON protocol. HeV was semi-quantitated using the cycling threshold (Ct) value for each dilution by RT-qPCR, with a panel of 3 samples selected, representing high, medium and low viral load (mean Ct; 27.0, 30.0, 33.0, respectively), with testing of 5 replicates of each of the sample cohorts of the panel.

*Stage 2: Assay Validation - Diagnostic Characteristics*

Validation of diagnostic characteristics of the HeV real-time RT-PCR assay involved processing of clinical specimens bio-banked within the BSL-4 laboratory at ACDP. A total of 74 samples were used in this study, consisting of 45 nasal swab and 29 whole blood specimens that had been collected from infected horses between 2009 and 2021 and previously tested positive for HeV by RT-qPCR (Feldman et al., 2009; Smith, Halpin, Warrilow, & Smith, 2001) Additionally, 25 nasal swabs and corresponding whole blood samples were obtained from vaccinated horses and horses previously excluded from HeV infection as part of previous laboratory diagnostic investigations. These samples were collected from The University of Queensland Equine Farm and tested by the Biosecurity Sciences Laboratory, Biosecurity Queensland, Department of Primary Industries. The combined dataset was used to estimate DSe and DSp using a Bayesian model, described below. Sample processing involved aliquoting half the sample and inactivating via the HUDSON protocol, with the remaining sample purified with the MagMax Pathogen RNA/DNA Kit for comparison of both sample preparation methods with the reference standard RT-qPCR (Wang et al., 2021).

### 2.6 Evaluation of diagnostic test characteristics using bayesian latent class modelling

Samples were classified as positives and negatives based on the Ct value cut-off threshold derived for the RT-qPCR assay assessed in this study. The RT-qPCR test currently used at the ACDP for molecular diagnostics was used to test the bio-banked samples (Wang et al., 2021). This was used as an ‘imperfect’ reference test (**Test 1; ACDP-PCR**) with RT-qPCR Ct threshold value of <40 indicating a positive result (Feldman et al., 2009; Smith et al., 2001). The results of the POC HeV RT-qPCR (Wang et al., 2021) assay run with purified RNA isolated from corresponding samples (**Test 2; PURIFIED RNA POC-RT-qPCR**) or crude lysate samples (diluted 1:10 in sample inactivation buffer and incubated at 95°C for 5 min); **Test 3; HUDSON POC-RT-qPCR**), were compared with the reference Test 1: ACDP-PCR to estimate the relative diagnostic test parameters (DSe and DSp). An RT-qPCR Ct threshold of <40.0 was used to classify positive samples for both Test 2 and Test 3 (Wang et al., 2021).

For estimating the relative DSp with 95% confidence intervals and an absolute precision of ± 2%, assuming a sensitivity of the modified RT-qPCR (Wang et al., 2021) of >90% and specificity to be >95%, the minimum required sample size was estimated *a priori* to be 864 samples for estimating DSe and 456 samples for estimating DSp (WOAH, 2023b). Since sufficient positive reference samples were not available for such an analysis, which made no assumption on the true disease status of the animals from which the samples were derived, a Bayesian latent class analysis was implemented, as recommended by WOAH (Cheung et al., 2021). The limited number of archived HeV positive clinical samples reflects both the episodic nature of HeV spillover events and the requirement for all confirmatory diagnostic work to be performed under PC4 containment, which restricts the availability of usable materials for downstream validation. Such constraints are well-recognised in the validation of assays for high-consequence pathogens. For this reason, Bayesian latent class modelling (BLCM) was adopted in accordance with WOAH recommendations as an appropriate alternative when sample numbers are insufficient for classical frequentist validation and when no perfect reference standard is available.

A Bayesian latent class model (BLCM) based on a publication by (Branscum, Gardner, and Johnson, 2005) was fitted with the assumption that all three tests were molecular tests detecting HeV RNA and were conditionally dependent. A 2-test in 2-population BLCM that allowed for DSe and DSp dependence between tests was fitted with covariances defined for positives and negatives (Dendukuri & Joseph, 2001). Since there were 8 parameters in the model (2 DSe and 2 DSp for the two tests, 2 covariances [for conditional dependencies for positive and negative results], and 2 population prevalences) to be estimated, there were only 6 degrees of freedom and hence, the model is non-identifiable (i.e. there is no unique set of parameters that generated the counts of pairwise test results (T1+T2+, T1+T2−, T1−T2+, T1−T2−) without informative prior information on at least 2 parameters. Incorporating scientifically justifiable priors for 2 parameters distinguishes a plausible solution from an infinite number of solutions when the model is non-identifiable. The dbeta (a, b) priors for the DSe and DSp for the ACDP-PCR were based on the estimates of DSe and DSp mentioned in (Daniels, Ksiazek, and Eaton, 2001) and were calculated in Betabuster 1.0 (https://betabuster.software.informer.com/). We also checked the posterior distributions with two sets of less informative priors (Table 3) to verify the results.

**Table 3:** Prior distributions for DSe and DSp of three molecular tests for diagnosing HeV infection in horses.

Test	DSe			DSp		
	Mode	5th percentile	Beta (a,b) distribution parameters	Mode	5th percentile	Beta (a,b) distribution parameters
**1. ACDP RT-qPCR**						
*Informative priors*	0.90	0.80	42.57, 5.62	0.95	0.90	99.70, 6.19
*Less informative priors 1*	Uniform		1, 1	Uniform		1, 1
*Less informative priors 2*	0.90	0.70	15.03, 2.56	0.95	0.80	21.20, 2.06
**2. PURIFIED RNA POC-RT-qPCR**	Uniform		1, 1	Uniform		1, 1
**3. HUDSON POC-RT-qPCR**	Uniform		1, 1	Uniform		1, 1
**Population**	**Mode, 5th/95th percentile and Beta(a, b) distribution parameters**					
*Horses from the University of Queensland farm (p1)*	0.001	0.01	1.36, 364.91			
*Samples from ACDP biobank (2009-2021; p2)*	0.90	0.80	42.57, 5.62			

Finally, a Bayesian latent class model for assessing the diagnostic test parameters for all three tests with the 2 populations was developed with conditional dependency between all three tests. Since all three were molecular assays based on RT-qPCR, they were conditionally dependent, and covariance and cross-covariance terms were added between the Se and Sp of these tests (Denis-Robichaud et al., 2024; Lurier et al., 2021).

Flat beta (1,1) priors were assumed for the DSe and DSp for the tests under evaluation (2. PURIFIED RNA POC-RT-qPCR and 3. HUDSON POC-RT-qPCR), given no available prior information. Samples from population 1 (p1) were collected from horses in the University of Queensland farm and were considered free from HeV infection. We considered zero prevalence of the disease in this population (p1). Since a beta distribution does not have mass over zero, we modelled true prevalence (π) = π* x τ, assumed probability tau (τ) from a Bernoulli distribution and a probability τ0 if π = 0. With the assumption that prevalence was 95% sure to be <0.01 with mode = 0.001, the uncertainty about the unknown π* was modelled as π*∼beta (aπ, bπ). The positive reference samples (population 2; p2) were collected over time and did not come from one geographical area. Based on expert opinion, we were 95% sure that 80 per cent would return a positive result with mode = 90 per cent. Scripts were run using OpenBUGS v3.2.3 (Lunn, Spiegelhalter, Thomas, & Best, 2009) with convergence estimates derived using 1,000,000 iterations of simulation to ensure the Monte Carlo (MC) error value was <5% of the standard deviation of the node estimate using three assumed chains as initials or three generated initials, and discarding 50,000 iterations as burn-in. Convergence was assessed by evaluating the history, trace plots and calculation of the Gelman–Rubin statistic diagnostic (Vats & Knudson, 2021), which compares the within and between chain variability of the three-run chains. Posterior medians with 95% probability intervals (PI) corresponding to the 2.5^th^ and 97.5^th^ percentiles of the MC sample were used to summarise parameter estimates of DSe, DSp, and apparent prevalence in the two populations (Balkema-Buschmann et al., 2022).

To complement estimates of DSe and DSp, receiver operating characteristic (ROC) curves were generated using MedCalc® Statistical Software version 23.1.3 (MedCalc Software Ltd, Ostend, Belgium; https://www.medcalc.org; 2025) and used to evaluate the diagnostic accuracy of HUDSON POC-RT-qPCR compared with PURIFIED RNA POC-RT-qPCR. The ROC curve plots the true positive rate (y‐axis) against the false positive rate (x‐axis), thereby assessing the reliability of true negative and positive results for HeV detection using HUDSON prepared clinical samples. The area under the ROC curve (AUC) was used to evaluate the impact of the HUDSON method on samples to detect HeV via RT-qPCR and ranges in values between 0.5 and 1. A perfect test has an AUC of 1 and an inefficient test has an area of 0.5. Based on arbitrary criteria, the following guidelines have been suggested for interpretation of intermediate AUC values: low (0.5 <, AUC ≤ 0.7), moderate (0.7 <, AUC ≤ 0.9), or high (0.9 < AUC ≤ 1) accuracy (Swets, 1988). Significant difference of the ROC curve from the reference line was assessed as P < 0.05.

*Stage 3: Assay Validation – Reproducibility*

Due to the biosafety restrictions associated with HeV and the limited number of approved facilities authorised to handle spiked materials in Australia, this reproducibility assessment represents a preliminary two-laboratory evaluation rather than a full WOAH recommended multi-laboratory study.

Preliminary assessment of reproducibility was assessed by testing of spiked HeV g1 and g2 gamma irradiated isolates in inactivation buffer. Five different runs were performed by two operators in two different laboratories (Biosecurity Sciences Laboratory [BSL], Biosecurity Queensland, Department of Primary Industries and School of Veterinary Science, University of Queensland) on 5 consecutive days with 5 replicates of a high, medium and low analyte concentration. Assays were evaluated by estimating the percent Coefficient of Variation (CV%) of replicates.

### 2.7 Ethics statement

No animal procedures were performed for the purposes of this study. All samples utilised were previously collected as part of routine diagnostic investigations or approved research activities and were accessed as biobanked material. The study was conducted under The University of Queensland administrative approval ANFRA (approval not requiring full application - Approval No. 2020/AE000174).

## 3 Results

### 3.1 Hendra virus positive sample inactivation: proof of concept

**Fig. 1:**
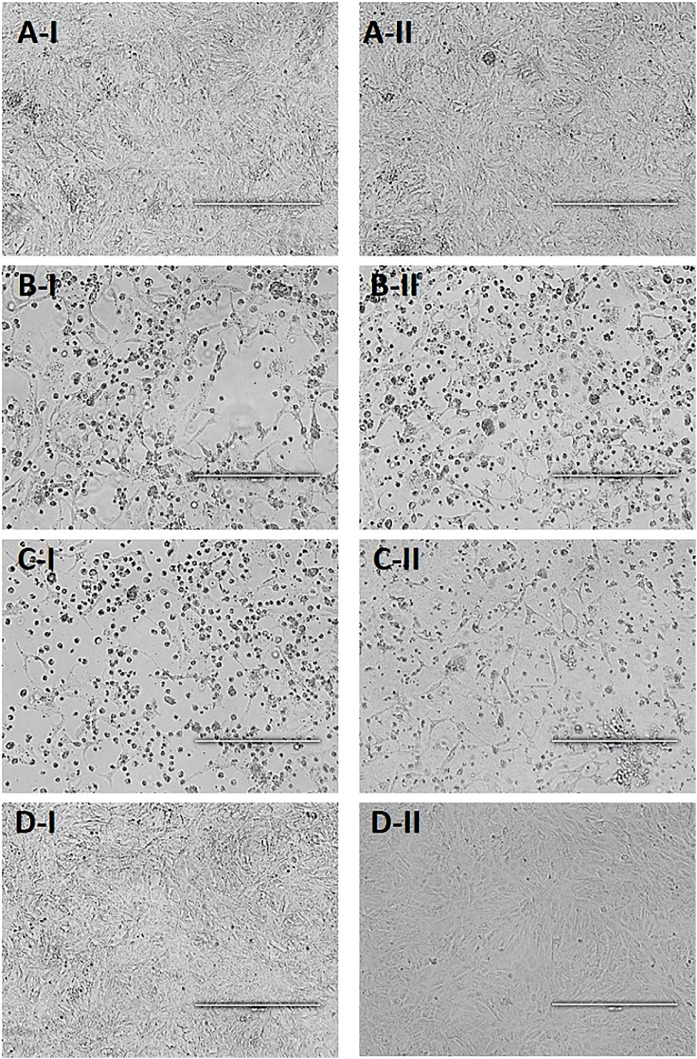
Cytopathic effect (CPE) following inoculation of Vero cells with HeV spiked whole blood treated with inactivation buffer (A-I to C-I) and HeV spiked swab treated with inactivation buffer (A-II to C-II). Representative images of Vero cells inoculated with (A) Sample incubated at 95°C for 5 min; (B) sample incubated at room temperature for 5 min; (C) positive control: HeV spiked PBS; and (D) negative control (untreated sample inactivation buffer) incubated at room temperature for 5 min.

The HUDSON heat inactivation method was assessed by using whole blood or nasal swab specimens (in virus transport medium), collected from healthy horses spiked with 1 × 10^4^ TCID_50_ of HeV and treated with sample inactivation buffer at 95°C for 5 min. Controls included HeV spiked sample diluted in PBS and untreated inactivation buffer incubated at room temperature for 5 min. As shown in Figure 1 A-I and A-II, no cytopathic effect (CPE) was observed in cells inoculated with the 95°C treated samples, whereas CPE was present in the control samples. RT-qPCR of culture supernatant was also negative, confirming HeV inactivation (Table 4).

*Stage 1: Assay Validation - Analytical Characteristics*

**Table 4:** RT-qPCR results of purified inoculum and culture supernatant following final pass to assess viral RNA integrity post treatment.

Sample	Sample Incubation	Inoculum (Mean Ct ± SD)	Final passage culture supernatant (Mean Ct ± SD)
HeV spiked equine nasal swab treated with inactivation buffer	95°C for 5 min	25.7 ± 0.2	NEG
HeV spiked equine nasal swab treated with inactivation buffer	RT for 5 min	23.3 ± 0.2	16.4 ± 0.2
HeV spiked equine nasal swab treated with PBS	RT for 5 min	22.9 ± 0.6	16.8 ± 0.8
HeV spiked equine whole blood treated with inactivation buffer	95°C for 5 min	23.4 ± 0.2	NEG
HeV spiked equine whole blood treated with inactivation buffer	RT for 5 min	22.3 ± 0.1	16.0 ± 0.1
HeV spiked equine whole blood treated with PBS	RT for 5 min	22.1 ± 0.2	15.7 ± 0.1
Negative control	RT for 5 min	NEG	NEG

*RT – Room temperature

### 3.2 Performance of the DARQ RT-LAMP Assay

**Fig. 2:**
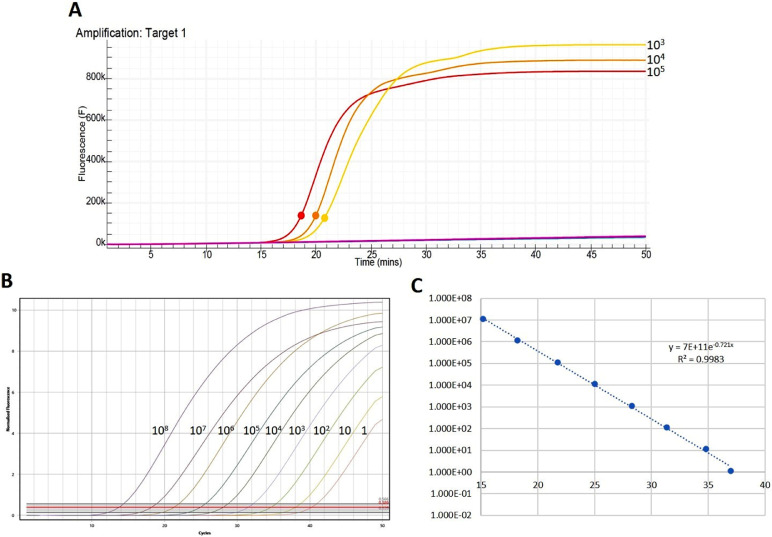
Limit of detection of DARQ RT-LAMP (A) and RT-qPCR; (B) using 10-fold serial dilutions of HeV P-gene (A) and M-gene (B), respectively; (C) HeV M-gene plasmid standard curve and amplification efficiency.

ASp of the HeV duplex DARQ RT-LAMP assay was assessed using purified viral and bacterial strains or isolates and demonstrated no cross-reactivity with non-target reference strains and isolates (Supp. Table 1). Using serial dilutions of each HeV gene plasmid, ASe testing determined a LOD of 10³ copies/µL for the DARQ RT-LAMP assay (Figure 2A).

As DARQ RT-LAMP was included as a preliminary feasibility assessment, these analytical findings were used to determine whether the assay met the minimum sensitivity benchmark required for progression. Although DARQ RT-LAMP successfully amplified HeV targets under controlled laboratory conditions, its ASe was insufficient for practical POC application. For high-consequence pathogens such as HeV, where early detection and risk mitigation are critical, POC assays must achieve ASe approaching that of reference RT-qPCR methods to reliably detect low viral loads present during early or subclinical infection. The three-log reduction in sensitivity observed for DARQ RT-LAMP relative to RT-qPCR therefore falls below the performance threshold required for safe and effective preliminary clinical screening. Consequently, further validation of this platform was discontinued, and DARQ RT-LAMP was not taken forward to DSe/DSp, reproducibility testing or Bayesian modelling.

### 3.3 Performance of the RT-qPCR assay

In parallel, RT-qPCR demonstrated substantially higher ASe, with an LOD of 1 copy/µL, representing a three-log improvement compared with DARQ RT-LAMP (Figure 2B). The standard curve generated using 10-fold serial dilutions of the HeV M-gene plasmid was linear across eight orders of magnitude (10⁸–10⁰ equivalent gene copies) (Figure 2C).

Analytical repeatability of the RT-qPCR assay was high, with inter-assay CV% ranging from 1.6% to 4.2% for HeV g1 and 1.1% to 2.3% for HeV g2 (Supp. Table 2). Intra-assay CV% for HeV g1 ranged from 1.0% to 2.0%, with between-run and between-operator CV% of 2.0% and 2.7%, respectively. For HeV g2, intra-assay CV% ranged from 0.7% to 1.7%, with between-run and between-operator CV% of 4.7% and 2.0%, respectively (Supp. Table 3).

*Stage 2: Assay Validation - Diagnostic Characteristics*

Since the ASe of DARQ RT-LAMP was inferior to the RT-qPCR, the assay failed to detect 42% of positive clinical samples (data not shown) using a subset of biobanked samples (N = 24). These samples had been previously confirmed as HeV-positive by the reference ACDP RT-qPCR assay (Feldman et al., 2009; Smith et al., 2001), with Ct values <40 as per standard diagnostic criteria, as described in the Materials and Methods. To determine DSe and DSp of the HeV RT-qPCR (Wang et al., 2021) by a Bayesian LCA, results from testing 74 HeV-positive equine clinical specimens (45 nasal swabs and 29 whole blood) and 25 HeV negative nasal swabs and paired whole blood samples from vaccinated horses were used.

### 3.4 Relative diagnostic test sensitivity (DSe) and diagnostic test specificity (DSp) of RT-qPCR assay by BLCM approach

Posterior distributions for DSe and DSp of the three tests are summarised in Table 5. Medians of the posterior DSe distributions were generally lower for the HUDSON POC-RT-qPCR [0.670 (0.557-0.770)] compared to the PURIFIED RNA POC-RT-qPCR [0.847 (0.752-0.917)] and the reference ACDP RT-qPCR [0.955 (0.908-0.983)]. The DSp for the two tests under evaluation (HUDSON POC-RT-qPCR and PURIFIED RNA POC-RT-qPCR) had identical DSp estimates [(0.978 (0.915-0.999)], which were comparable between the reference ACDP RT-qPCR [0.959 (0.921-0.983)]. All three assumed models converged after 20,000 iterations, irrespective of the priors for sensitivity and specificity (Supp. Table 4).

**Table 5:** Posterior distributions (medians and 95% probability intervals) for DSe and DSp of RT-qPCR tests for diagnosing HeV infection in horses by prior distribution source and sample types. ^ posterior estimates when reference test used with HUDSON POC-RT-qPCR; ^^ posterior estimates when reference test used with PURIFIED RNA POC-RT-qPCR.

Test and samples	DSe (95% PI)				DSp (95% PI)		
	Informative priors	Less informative priors 1	Less informative priors 2	Informative priors		Less informative priors 1	Less informative priors 2
**ACDP RT-qPCR**							
*Swabs*	0.942 (0.880-0.978)^ 0.941 (0.880-0.978)^^	0.984 (0.919-0.999)^ 0.984 (0.919-0.999)^^	0.963 (0.895-0.993)^ 0.963 (0.895-0.993)^^	0.952 (0.906-0.980)^ 0.952 (0.906-0.981)^^		0.963 (0.87-0.999)^ 0.963 (0.838-0.999^^	0.956 (0.871-0.993)^ 0.956 (0.871-0.993)^^
*Whole blood*	0.930 (0.857-0.973)^ 0.928 (0.853-0.973)^^	0.976 (0.879-0.999)^ 0.975 (0.872-0.999)^^	0.950 (0.861-0.990)^ 0.948 (0.854-0.990)^^	0.952 (0.906-0.981)^ 0.952 (0.906-0.981)^^		0.964 (0.841-0.999)^ 0.964 (0.841-0.999)^^	0.957 (0.871-0.993)^ 0.956 (0.873-0.993)^^
*All samples*	0.955 (0.907-0.983)^ 0.955 (0.908-0.983)^^	0.990 (0.949-1.000)^ 0.990 (0.949-1.000)^^	0.971 (0.926-0.995)^ 0.974 (0.926-0.995)^^	0.959 (0.920-0.983)^ 0.959 (0.921-0.983)^^		0.982 (0.915-0.999)^ 0.982 (0.916-0.999)^^	0.971 (0.914-0.995)^ 0.972 (0.914-0.995)^^
**HUDSON POC-RT-qPCR**							
*Swabs*	0.631 (0.488-0.761)	0.636 (0.490-0.766)	0.634 (0.489-0.763)	0.965 (0.859-0.999)		0.966 (0.849-0.999)	0.966 (0.856-0.999)
*Whole blood*	0.713 (0.542-0.851)	0.724 (0.548-0.861)	0.718 (0.546-0.856)	0.965 (0.859-0.999)		0.967 (0.852-0.999)	0.966 (0.855-0.999)
*All samples*	0.670 (0.557-0.770)	0.675 (0.562-0.777)	0.673 (0.561-0.774)	0.978 (0.915-0.999)		0.983 (0.920-0.999)	0.981 (0.917-0.999)
**PURIFIED RNA POC-RT-qPCR**							
*Swabs*	0.804 (0.676-0.902)	0.818 (0.688-0.911)	0.812 (0.684-0.907)	0.965 (0.859-0.999)		0.967 (0.851-0.999)	0.966 (0.856-0.999)
*Whole blood*	0.883 (0.742-0.9733)	0.895 (0.754-0.975)	0.888 (0.746-0.973)	0.965 (0.860-0.999)		0.966 (0.851-0.999)	0.966 (0.856-0.999)
*All samples*	0.847 (0.752-0.917)	0.858 (0.766-0.926)	0.853 (0.760-0.922)	0.978 (0.915-0.999)		0.983 (0.919-0.999)	0.981 (0.917-0.999)

^ vs HUDSON POC-RT-qPCR

^^ vs PURIFIED RNA POC-RT-qPCR

When all samples were considered with informative prior distributions for sensitivity and specificity, the posterior distributions for the true prevalence of HeV infection in population 1 (The University of Queensland Farm horses) were 0.004 (95% probability interval, 0.000–0.011) and in population 2 (ACDP bio-bank samples) 0.99 (0.877-0.999). These were comparable when less informative prior distributions 1 and 2 were used. Results of the BLCM assuming conditional dependence between all three tests are presented in Supp. Table 5.

The posterior estimates of the reference test ACDP RT-qPCR did not differ between models comparing the tests under evaluation (HUDSON POC-RT-qPCR and PURIFIED RNA POC-RT-qPCR) but differed based on different prior distributions. The less informative priors 1 distribution overestimated the DSe and DSp of all three tests, while the estimates obtained using the informative and less informative prior 2 did not differ.

### 3.5 Receiver-operating characteristic (ROC) analysis for comparing sample preparation methods

**Fig. 3:**
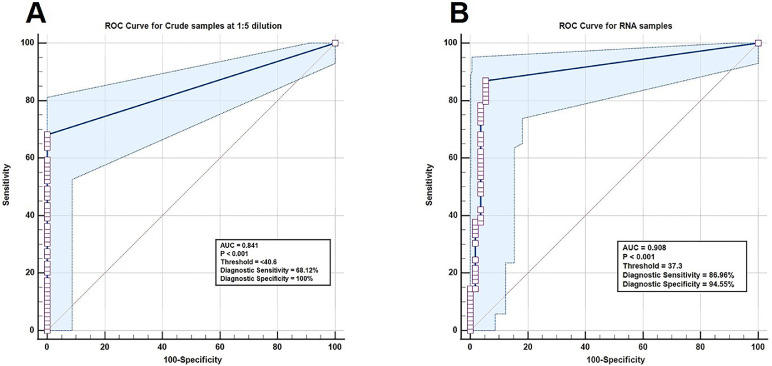
Receiver operating characteristic curves for (A) HUDSON POC-RT-qPCR; (B) PURIFIED RNA POC-RT-qPCR.

ROC curves for HeV HUDSON POC-RT-qPCR and PURIFIED RNA POC-RT-qPCR showed that the HUDSON POC-RT-qPCR had an AUC of 0.841 (P = <0.001; Fig. 3A) and PURIFIED RNA POC-RT-qPCR had an AUC of 0.908 (P <0.001; Fig. 3B)

*Stage 3: Assay Validation – Reproducibility*

Preliminary reproducibility results from testing a limited number of samples in two laboratories indicated no statistically significant differences of the tests between operators, laboratories and analyte concentration in the HeV g1 sample (Supp. Table 3). However, for HeV g2, the CV% for the sample with low viral load concentration differed significantly (Student t test; P<0.001) between operators by approximately one Ct. The tests had high reproducibility within operators, between runs and days where the CV% was <10% for both HeV g1 and g2.

## 4 Discussion

Hendra virus is a highly lethal zoonotic pathogen that poses a serious public and veterinary health concern in eastern Australia. This study applied a science based validation framework to assess key performance parameters relevant to POC molecular diagnostic platforms for the rapid detection of HeV in horses. Using established validation guidance, we evaluated the analytical and diagnostic performance of two candidate testing platforms and estimated diagnostic sensitivity and specificity in equine samples from infected and non-infected populations using Bayesian latent class modelling.

The results of this study demonstrate that the RT-qPCR platform, particularly when combined with rapid HUDSON sample preparation, is suitable for use as a preliminary POC molecular screening assay for HeV in equine clinical settings. While diagnostic sensitivity was reduced in HUDSON treated samples compared with purified RNA, the consistently high diagnostic specificity supports its use as a rule-in or exclusion decision support tool during the early case assessment. Confirmatory testing using reference laboratory RT-qPCR remains essential for definitive diagnosis and regulatory reporting.

An important component of the POC HeV tests evaluated in this study was the incorporation of the HUDSON method for sample preparation, which enables viral lysis and inactivation through combined heat and chemical treatment (Myhrvold et al., 2018). We demonstrated that HUDSON effectively inactivates HeV while preserving RNA suitable for downstream RT-qPCR, thereby eliminating the need for RNA purification procedures. Studies have demonstrated the HUDSON method has been particularly valuable in settings where rapid diagnostics are crucial, such as in the field or in low-resource environments, and has been used in detecting a range of pathogens, including Zika, Dengue, Ebola and Lassa viruses (Barnes et al., 2020; Myhrvold et al., 2018).

Evaluation of the DARQ RT-LAMP assay showed a limit of detection of 1,000 copies/µL, comparable to previously reported HeV POC isothermal amplification assays reported by (Foord, Middleton, and Heine, 2012); (Pollak, Marsh, Olsson, McMillan, and Macdonald, 2023); with no cross-reactivity observed against non-target reference strains. However, this level of ASe was insufficient for its intended application and therefore, not fit for purpose. The reduced analytical sensitivity observed for DARQ RT-LAMP may reflect the combined effects of multiplex assay design and potential template fragmentation associated with gamma-irradiated material, which may disproportionately impact amplification efficiency in assays requiring multiple primer binding sites such as DARQ-RT-LAMP. In contrast, parallel evaluation of a POC HeV RT-qPCR laboratory-based assay (Wang et al., 2021) demonstrated substantially ASe by three decimal dilutions, equivalent to a detection limit of 1 copy/µL. To date, the HeV RT-qPCR assessed in this study is currently the most sensitive diagnostic assay when compared to previous reports (Foord et al., 2012; Pollak et al., 2023; Wang et al., 2021).

Considering the assay sensitivity limitations observed with the HeV DARQ RT-LAMP platform, the focus of the study shifted from comparison of POC testing platforms, to further evaluation of the HeV RT-qPCR-based POC diagnostic test, which offered improved sensitivity and the potential for field application. Based on the BLCM estimates, the DSe of the HUDSON POC-RT-qPCR (67.0%) was considerably lower when compared to the purified RNA POC-RT-qPCR assay (84.7%) and the reference test (95.5%; ACDP RT-qPCR). However, the high diagnostic specificities of all three tests were comparable and the posterior intervals overlapped. It can, therefore, be concluded, that the HUDSON POC-RT-qPCR assay can be used as a preliminary assessment to rule in disease given its high DSp (>0.96%), which ensures that positive samples are likely true positives. Confirmatory testing should still be conducted at a reference laboratory to validate results from the field or veterinary clinic. Additionally, the high DSp (97.8%) of the HUDSON POC-RT-qPCR results in a very low false positive rate, making it particularly valuable for indicating infection, as positive results strongly indicate true HeV cases.

A key constraint in this validation was the limited availability of HeV positive clinical specimens, which is a recognised challenge when working with high-consequence pathogens requiring PC4 containment. The resulting dataset (74 positive and 25 negative archived samples) falls below WOAH recommendations for full validation; however, these constraints reflect unavoidable biosafety and epidemiological limitations rather than study design. To ensure scientific robustness despite small sample numbers, we employed a BLCM framework in accordance with WOAH guidance for situations where perfect reference tests or large datasets are unavailable. BLCM allowed incorporation of prior biological knowledge, explicit modelling of test dependencies and estimation of DSe and DSp with defined uncertainty intervals. While wider posterior intervals are an expected outcome of such constraints, the modelling approach provides a valid and internationally accepted means of assessing test performance in the context of limited specimen availability. Further prospective studies incorporating additional naturally occurring cases will help refine these estimates as more samples become accessible.

Improving the DSe of the HUDSON POC-RT-qPCR could be achieved through beta-testing in a facility such as The University of Queensland, where samples collected from vaccinated horses can be prepared in-clinic and then sent to an on-site diagnostic laboratory for confirmation using nucleic acid extracts. Running both the HUDSON and extracted samples in parallel using RT-qPCR would allow for a direct comparison and potential refinement of the DSe of the HUDSON POC-RT-qPCR.

The successful adaptation of the laboratory-based HeV RT-qPCR test for POC platforms demonstrates the feasibility of integrating advanced molecular diagnostics into field applications, bridging the gap between laboratory accuracy and field practicality. Due to the lower LOD of 1 copy /ul and higher DSe of 95.5% (95%PI 90.81%-98.3%) and equally high DSp of 97.8% (95%PI 91.5%-99.9%) compared to the DARQ RT-LAMP assay, the RT-q-PCR showed the better fitness for purpose to detect HeV in horses with an acute infection.

To obtain a preliminary assessment of reproducibility, samples spiked with high, medium and low levels of gamma-irradiated HeV culture were tested by two operators at different laboratories on different days with 5 replicates of each sample. Although WOAH recommends a minimum of 3 laboratories and 20 samples to assess reproducibility, biosafety and logistical constraints in this study limited the number of laboratories/operators and samples involved. The high reproducibility (CV <10%) indicated satisfactory robustness and ruggedness of the POC when testing samples of different analyte concentration at different sites and operators. Further assessments in the field under different environmental circumstances is necessary to corroborate these preliminary results.

The development of the HeV HUDSON RT-qPCR POC test has significant implications for the equine industry and public health. Rapid and accurate diagnosis of HeV infections in horses is crucial for implementing timely interventions, reducing the risk of transmission to humans, and ensuring the welfare of affected animals. The Mic PCR platform's portability and ease of use make it a valuable tool for appropriately trained veterinarians and biosecurity personnel, enabling prompt and informed decision-making in the field.

While the portability of the Mic PCR platform and the simplicity of the HUDSON workflow offer clear advantages for POC use, the practical application of a molecular assay in equine settings requires consideration of several operational factors. As with any molecular method, appropriate user training, attention to sample handling and routine quality assurance are necessary to ensure reliable results. Accordingly, the HUDSON RT-qPCR assay is best positioned as a rapid preliminary test to support early veterinary decision making while laboratory confirmation is pending. Further assessment of assay performance under field conditions, including testing by end-users in a range of operational environments, will help define practical boundaries of use and guide integration into routine equine biosecurity workflows. These steps align with the staged nature of diagnostic test validation recommended by WOAH, whereby operational feasibility and ruggedness are strengthened progressively as analytical and diagnostic characteristics are established.

The integration of the HeV HUDSON RT-qPCR POC test into existing biosecurity protocols can enhance overall disease management strategies. By providing a reliable diagnostic tool that can be used directly in the field, the risk of widespread outbreaks can be mitigated, and the impact on equine training and breeding facilities minimized. In veterinary workflows, the HUDSON RT-qPCR assay is positioned to function as an early decision-support tool during suspected HeV incidents. Following sample collection under standard biosecurity precautions, the HUDSON inactivation step enables safe handling at the point of care, after which the portable Mic PCR platform can generate preliminary results within approximately one hour. These results assist veterinarians in making immediate decisions regarding case management, isolation, movement restrictions and personal protective equipment, while confirmatory laboratory RT-qPCR testing proceeds through established reporting pathways. Standard operating procedures and targeted training can support consistent implementation across field and clinic settings as adoption expands. Furthermore, the availability of such a diagnostic tool can support ongoing vaccination efforts by facilitating timely monitoring and control of HeV infections.

Adoption of this rapid and accurate HeV detection method could facilitate earlier intervention, mitigating risks to both equine and human populations and supporting the ongoing efforts to control HeV outbreaks in Australia. Collaboration with regulatory bodies and stakeholders in the equine industry will be essential to ensure appropriate reporting pathways and the successful adoption of this diagnostic tool. Continued training for end-users, particularly veterinarians, will be crucial to maximise the test's utility and effectiveness in the field. Molecular platforms are technically more complex than lateral flow tests and require high levels of training and competency. Effective use of these platforms in field or clinical settings depends not only on user proficiency but also on strict adherence to quality assurance practices. Training should therefore cover sample handling, assay setup, interpretation of results and troubleshooting, supported by quality assurance measures such as internal controls, standardised protocols and participation in external quality assessment programmes. Ensuring these standards are met is critical for reliable POC molecular diagnostics and timely, dependable results in real-world settings.

## Funding

This project was supported by AgriFutures Australia [grant numbers PRJ-012093 and FRP-015556] as part of the AgriFutures Thoroughbred Horses Program.

**Abbreviations**
ACDP: Australian Centre for Disease Preparedness
ASe: Analytical sensitivity
ASP: Analytical specificity
ATCC: American Type Culture Collection
AUC: Area under the curve
BLAST: Basic Local Alignment Search Tool
BLCM: Bayesian latent class modelling
BSL: Biosafety level
CPE: Cytopathic effect
Ct: Cycling threshold
CV: Coefficient of variation
DARQ: Detection amplification by release of quenching
DSe: Diagnostic sensitivity
DSp: Diagnostic specificity
DNA: Deoxyribonucleic Acid
dNTP: Deoxynucleoside Triphosphate
EDTA: Ethylenediaminetetraacetic acid
EMEM: Eagle’s Minimal Essential Medium
FBS: Foetal Bovine Serum
HEPES: N-2-hydroxyethylpiperazine-N'-2-ethanesulfonic acid
HeV: Hendra virus
HUDSON: Heating unextracted diagnostic samples to obliterate nucleases
KCl: Potassium Chloride
LAMP: Loop-mediated isothermal amplification
LOD: Limit of detection
NaOH: Sodium Hydroxide
mtDNA: Mitochondrial DNA
MWCO: Molecular Weight Cut-Off
PBS: Phosphate buffered saline
PC4: Physical containment level 4
PCR: Polymerase chain reaction
POC: Point-of-care
RNA: Ribonucleic Acid
ROC: Receiver-operating characteristic
rRNA: Ribosomal RNA
RT-LAMP: Reverse transcription loop-mediated isothermal amplification
RT-qPCR: Reverse transcription quantitative polymerase chain reaction
SCAHLS: Subcommittee on Animal Health Laboratory Standards
Tris-HCl: Tris hydrochloride
VTM: Virus transport medium
WOAH: World Organisation for Animal Health

## Ethical statement

This study was conducted in accordance with the Australian Code for the Care and Use of Animals for Scientific Purposes and was approved by the University of Queensland Animal Ethics Committee (Approval No. 2020/AE000174: ANRFA – Rapid point-of-care diagnosis of equine Hendra virus (HeV) using LAMP technology). All equine clinical specimens used in this study comprised previously collected and archived nasal swabs and whole blood samples obtained during routine diagnostic investigations. No animals were prospectively sampled or handled specifically for the purposes of this study and no additional procedures were performed on live animals. Use of archived specimens was covered under the existing ethics approval, and all samples were de-identified prior to analysis.

## CRediT authorship contribution statement

**Lyndal Hulse:** Writing – review & editing, Writing – original draft, Visualization, Validation, Resources, Methodology, Formal analysis, Conceptualization. **Leonard Izzard:** Writing – review & editing, Visualization, Validation, Resources, Methodology, Formal analysis. **Singanallur Balasubramanian Nagendrakumar:** Writing – review & editing, Writing – original draft, Visualization, Validation, Methodology, Formal analysis, Data curation. **Axel Colling:** Writing – review & editing, Validation, Resources. **Darren Underwood:** Writing – review & editing, Validation, Resources, Methodology. **Luke Driver:** Writing – review & editing, Validation, Resources, Methodology. **David T. Williams:** Writing – review & editing, Validation, Resources. **Benjamin Ahern:** Resources, Project administration, Funding acquisition, Conceptualization.

## Declaration of competing interest

The authors declare the following financial interests/personal relationships which may be considered as potential competing interests:

Dr Lyndal Hulse reports financial support was provided by AgriFutures. If there are other authors, they declare that they have no known competing financial interests or personal relationships that could have appeared to influence the work reported in this paper.

## Appendix Supplementary materials

Image, application 1

Image, application 2

Image, application 3

Image, application 4

Image, application 5
