# Supplementary material for: Evaluation of two point-of-care molecular diagnostic platforms for rapid detection of equine Hendra virus
Source: Vet Anim Sci. 2026 May 30;33:100713. doi: 10.1016/j.vas.2026.100713 (PMC13253132; doi:10.1016/j.vas.2026.100713)
Supplement: Supplementary file 1 [file mmc1.docx]

Supplementary Table 1. Specificity of the HeV duplex DARQ RT-LAMP and multiplex real-time RT-qPCR against target and non-target reference strains and isolates.

| **Strain/isolate** | **HeV duplex DARQ RT-LAMP** | | **Multiplex real-time RT-PCR** | |
| --- | --- | --- | --- | --- |
|  | **HeV** | **Internal control** | **HeV** | **Internal control** |
| Hendra virus – prototype sequence (g1) | Positive | Positive | Positive | Positive |
| Hendra virus – variant sequence (g2) | Positive | Positive | Positive | Positive |
| Influenza virus type A | -- | Positive | -- | Positive |
| Equine herpesvirus strain 1 | -- | Positive | -- | Positive |
| Equine herpesvirus strain 2 | -- | Positive | -- | Positive |
| ABLV insectivorous strain | -- | Positive | -- | Positive |
| Equine herpesvirus strain 4 | -- | Positive | -- | Positive |
| Equine herpesvirus strain 5 | **--** | Positive | **--** | Positive |
| Japanese encephalitis virus | **--** | Positive | **--** | Positive |
| Murray Valley encephalitis virus | **--** | Positive | **--** | Positive |
| *Streptococcus equi* subsp. *zooepidemicus* B4181 | **--** | Positive | **--** | Positive |
| *Strep. Dysgalactiae* sp. B1156 | **--** | Positive | **--** | Positive |
| *Rhodococcus equi* B4639 | **--** | Positive | **--** | Positive |
| *Strep. Equi* subsp. *Equi* B3857 | **--** | Positive | **--** | Positive |
| *Staph aureus* B4840 | **--** | Positive | **--** | Positive |
| *Actinobacillus equuli* subsp. *haemolyticus* B764 | **--** | Positive | **--** | Positive |
| *Klebsiella pneumoniae* B5112 | **--** | Positive | **--** | Positive |
| *Klebsiella oxytoca* B4773 | **--** | Positive | **--** | Positive |
| Elsey Virus | **--** | Positive | **--** | Positive |
| Ross River Virus | **--** | Positive | **--** | Positive |
| Leptospira h. | **--** | Positive | **--** | Positive |
| *Chlamydia psittaci* | **--** | Positive | **--** | Positive |
| Kunjin Virus | **--** | Positive | **--** | Positive |
