# Supplementary material for: Evaluation of two point-of-care molecular diagnostic platforms for rapid detection of equine Hendra virus
Source: Vet Anim Sci. 2026 May 30;33:100713. doi: 10.1016/j.vas.2026.100713 (PMC13253132; doi:10.1016/j.vas.2026.100713)
Supplement: Supplementary file 2 [file mmc2.docx]

Supplementary Table 2. HeV RT-qPCR inter-assay coefficient of variation (%).

| **HeV g1**  **Viral load** | **qPCR Cycling Threshold (Ct)** | | | | | | | | | |  |  |
| --- | --- | --- | --- | --- | --- | --- | --- | --- | --- | --- | --- | --- |
|  | **Duplicate 1** | | **Duplicate 2** | | **Duplicate 3** | | **Duplicate 4** | | **Duplicate 5** | |  |  |
|  | **HeV** | **IC** | **HeV** | **IC** | **HeV** | **IC** | **HeV** | **IC** | **HeV** | **IC** | **CV (%) HeV** | **CV (%) IC** |
| **High** | 32.99 | 19.14 | 32.61 | 19.59 | 32.09 | 19.26 | 33.12 | 19.68 | 31.99 | 20.35 | 1.6 | 2.4 |
| **Medium** | 36.95 | 20.04 | 37.79 | 23.89 | 37.34 | 20.04 | 34.38 | 20.68 | -- | 22.33 | 4.2 | 7.8 |
| **Low** | 35.34 | 19.84 | 35.18 | 20.05 | 37.31 | 19.15 | -- | 19.93 | 35.78 | 18.51 | 2.7 | 3.3 |
|  |  |  |  |  |  |  |  |  |  | Total | 2.9 | 4.6 |

| **HeV g2**  **Viral load** | **qPCR Cycling Threshold (Ct)** | | | | | | | | | |  |  |
| --- | --- | --- | --- | --- | --- | --- | --- | --- | --- | --- | --- | --- |
|  | **Duplicate 1** | | **Duplicate 2** | | **Duplicate 3** | | **Duplicate 4** | | **Duplicate 5** | |  |  |
|  | **HeV** | **IC** | **HeV** | **IC** | **HeV** | **IC** | **HeV** | **IC** | **HeV** | **IC** | **CV (%) HeV** | **CV (%) IC** |
| **High** | 28.26 | 19.33 | 28.35 | 17.92 | 28.74 | 18.52 | 28.7 | 19.25 | 28 | 19.12 | 1.1 | 3.2 |
| **Medium** | 31.01 | 20.03 | 30.67 | 19.74 | 31.8 | 18.55 | 31.16 | 20.02 | 31.01 | 18.9 | 1.3 | 3.5 |
| **Low** | 33.83 | 18.27 | 33.61 | 18.06 | 33.81 | 19.98 | 34.92 | 18.15 | 32.72 | 20.17 | 2.3 | 5.6 |
|  |  |  |  |  |  |  |  |  |  | Total | 1.6 | 4.1 |

* IC = Internal control
