# Supplementary material for: Evaluation of two point-of-care molecular diagnostic platforms for rapid detection of equine Hendra virus
Source: Vet Anim Sci. 2026 May 30;33:100713. doi: 10.1016/j.vas.2026.100713 (PMC13253132; doi:10.1016/j.vas.2026.100713)
Supplement: Supplementary file 5 [file mmc5.docx]

Supplementary Table 5A. Posterior estimates of the DSe and DSp of the three HeV molecular methods showing the mean, standard deviation, 95% credibility intervals (2.50 and 97.5 percentiles) and median values for samples in virus transportation medium (VTM).

|  | **Mean** | **SD** | **2.50%** | **Median** | **97.50%** |
| --- | --- | --- | --- | --- | --- |
| Prev1 | 0.895 | 0.029 | 0.832 | 0.898 | 0.946 |
| Prev2 | 0.018 | 0.015 | 0.001 | 0.015 | 0.056 |
| Se_ACDP-PCR | 0.936 | 0.020 | 0.893 | 0.937 | 0.969 |
| Se_UQ-PCR | 0.896 | 0.046 | 0.795 | 0.900 | 0.974 |
| Se_CRUDE-PCR | 0.725 | 0.073 | 0.575 | 0.727 | 0.862 |
| Sp_ACDP-PCR | 0.945 | 0.017 | 0.906 | 0.947 | 0.974 |
| Sp_UQ-PCR | 0.928 | 0.045 | 0.816 | 0.937 | 0.990 |
| Sp_CRUDE-PCR | 0.924 | 0.049 | 0.801 | 0.934 | 0.989 |
| covse_000 | -0.052 | 0.027 | -0.111 | -0.050 | -0.005 |
| covse_001 | 0.060 | 0.025 | 0.019 | 0.057 | 0.116 |
| covse_010 | -0.005 | 0.015 | -0.030 | -0.008 | 0.032 |
| covse_011 | -0.002 | 0.018 | -0.035 | -0.003 | 0.035 |
| covse_100 | 0.041 | 0.027 | -0.003 | 0.038 | 0.103 |
| covse_101 | -0.049 | 0.026 | -0.103 | -0.048 | -0.002 |
| covse_110 | 0.016 | 0.030 | -0.042 | 0.016 | 0.075 |
| covse_111 | -0.009 | 0.031 | -0.076 | -0.007 | 0.049 |
| covsp_000 | 0.033 | 0.023 | -0.010 | 0.032 | 0.082 |
| covsp_001 | -0.021 | 0.017 | -0.059 | -0.019 | 0.008 |
| covsp_010 | -0.021 | 0.019 | -0.063 | -0.019 | 0.013 |
| covsp_011 | 0.009 | 0.012 | -0.008 | 0.006 | 0.039 |
| covsp_100 | -0.023 | 0.017 | -0.057 | -0.023 | 0.008 |
| covsp_101 | 0.011 | 0.013 | -0.005 | 0.008 | 0.043 |
| covsp_110 | 0.012 | 0.015 | -0.009 | 0.009 | 0.048 |
| covsp_111 | 0.001 | 0.012 | -0.023 | 0.000 | 0.026 |

Supplementary Table 5B. Posterior estimates of the DSe and DSp of the three HeV molecular methods showing the mean, standard deviation, 95% credibility intervals (2.50 and 97.5 percentiles) and median values for blood samples in 10% EDTA buffer.

|  | **Mean** | **SD** | **2.50%** | **Median** | **97.50%** |
| --- | --- | --- | --- | --- | --- |
| Prev1 | 0.896 | 0.031 | 0.828 | 0.899 | 0.949 |
| Prev2 | 0.019 | 0.015 | 0.001 | 0.015 | 0.056 |
| Se_ACDP-PCR | 0.941 | 0.019 | 0.900 | 0.943 | 0.973 |
| Se_UQ-PCR | 0.927 | 0.048 | 0.812 | 0.936 | 0.995 |
| Se_CRUDE-PCR | 0.714 | 0.081 | 0.546 | 0.718 | 0.860 |
| Sp_ACDP-PCR | 0.945 | 0.017 | 0.906 | 0.947 | 0.974 |
| Sp_UQ-PCR | 0.930 | 0.044 | 0.821 | 0.938 | 0.991 |
| Sp_CRUDE-PCR | 0.930 | 0.044 | 0.820 | 0.939 | 0.989 |
| covse_000 | -0.031 | 0.025 | -0.086 | -0.029 | 0.013 |
| covse_001 | 0.010 | 0.015 | -0.012 | 0.006 | 0.047 |
| covse_010 | 0.021 | 0.024 | -0.016 | 0.018 | 0.076 |
| covse_011 | 0.000 | 0.018 | -0.033 | -0.001 | 0.039 |
| covse_100 | 0.037 | 0.029 | -0.008 | 0.034 | 0.104 |
| covse_101 | -0.016 | 0.026 | -0.071 | -0.014 | 0.032 |
| covse_110 | -0.028 | 0.030 | -0.092 | -0.026 | 0.025 |
| covse_111 | 0.007 | 0.029 | -0.051 | 0.006 | 0.067 |
| covsp_000 | 0.033 | 0.023 | -0.010 | 0.031 | 0.082 |
| covsp_001 | -0.020 | 0.017 | -0.058 | -0.018 | 0.008 |
| covsp_010 | -0.021 | 0.019 | -0.063 | -0.020 | 0.012 |
| covsp_011 | 0.009 | 0.012 | -0.007 | 0.006 | 0.039 |
| covsp_100 | -0.024 | 0.017 | -0.057 | -0.023 | 0.008 |
| covsp_101 | 0.011 | 0.013 | -0.005 | 0.008 | 0.043 |
| covsp_110 | 0.012 | 0.015 | -0.009 | 0.009 | 0.048 |
| covsp_111 | 0.000 | 0.012 | -0.024 | 0.000 | 0.026 |
